# Sustainable Packaging Material Based on PCL Nanofibers and *Lavandula luisieri* Essential Oil, to Preserve Museological Textiles

**DOI:** 10.3390/polym14030597

**Published:** 2022-02-02

**Authors:** Ester F. Ferreira, Cláudia Mouro, Lúcia Silva, Isabel C. Gouveia

**Affiliations:** 1 FibEnTech Research Unit, Faculty of Engineering, University of Beira Interior, 6200-001 Covilhã, Portugal; ester.ferreira@ubi.pt (E.F.F.); d1684@ubi.pt (C.M.); mlas@ubi.pt (L.S.); 2 Chemistry Department, Faculty of Sciences, University of Beira Interior, 6200-001 Covilhã, Portugal

**Keywords:** museum-textiles, polycaprolactone, *Lavandula luisieri*, electrospinning, bioactive packaging

## Abstract

The connection with textiles is one of the oldest traditions in humanity, and in the historical scenario, textiles and clothing deal with material culture. Therefore, preservation is of the utmost importance to keep this important heritage. Packaging and protection of museological textiles is imperative due to the risks that these articles suffer, mainly concerning the attack of microorganisms that promote the acceleration of their degradation, and it is still necessary to create a proper packing material. In the present work we describe a bibliographic review about the museological scenario, focused on the packaging for preservation of textile articles, as well as the techniques usually used in preventive material conservation. Future perpsctives for the improvement in the conservation of museological textiles are also given. This research aims to produce a sustainable material based on polycaprolactone (PCL), with and without antimicrobial function by incorporating *Lavandula luisieri* essential oil (EO), in the form of a non-woven substrate for museological packaging. A comparison was made with the most frequently used materials, such as raw cotton and a non-woven polyester. The results demonstrated that both PCL and PCL + EO obtained a good characterization for museological application with good breaking strength and excellent whiteness index. In addition, PCL + EO showed a high bacterial reduction when compared with other protective materials frequently used in museums. Therefore, these findings emphasize the potential use of this material as an innovative protective antibacterial museological packaging solution, able to safeguard and preserve textile museum and clothing collections for longer and for future generations.

## 1. Introduction

Nowadays people’s awareness has grown about the need to document, conserve, and preserve the knowledge acquired since ancient times, in particular about textile articles, such as fibers, yarns, fabrics, knits, embroidery, and lace, among others that are in several museums, to establish the relationship between clothing and history helping to understand the culture of a particular people or period [1].

One of the main factors that justifies this research is the report that the amount of textile objects in museum collections is extremely small in comparison with objects of other materials, due to the difficulty of safeguarding these items which requires delicate conservation processes and complex actions in cases of restoration, in addition to the fact that textiles suffer natural and easy deterioration [2].

The acceleration of textile degradation occurs through the growth of microorganisms since textile substrates give the carbon source for these organisms. Certain environmental conditions in museums contribute to this situation, as does the greater fragility of textiles of natural-origin, such as cotton, wool, silk, and linen that constitute the major part of museum collections [3,4].

The action of microorganisms causes undesirable changes in textile articles, such as stains, reduced structural strength, and color changes, or even complete degradation of the material [5]. Another issue that hinders the work of institutions is related to the difficult selection of a suitable antimicrobial for textiles since each fiber has a different composition. In addition, there is a wide range of microorganisms that attack the fibers and a wrong choice of antimicrobial can accelerate further the degradation of the material [6,7].

To accomplish conservation of textiles, different methods have been used, such as chemical disinfection using alcohols, quaternary ammonium compounds, essential oils, among others; and physical disinfection by high and low temperature, pressure, modified atmospheres, and irradiation has also been used. However, these methods present limitations, such as changes in pH, color, or structure, as well as accelerated aging, or pose a threat to human health, or do not allow treatment of large numbers of items [5].

Currently, the preservation of museological textiles remains a challenge for conservators, microbiologists, chemists, textile scientists, and other specialists, and the decontamination of infected materials can lead to great costs for institutions. Thus, it is necessary to invest in new ways to safeguard this important part of cultural heritage [8]. For this reason, professionals began to identify that preventive conservation is as important as active conservation, as certain treatments can accelerate the deterioration of museum articles [6].

Preventive conservation guidelines for museums require stable and adequately ventilated microclimatic conditions, with values such as 20 °C for temperature and relative humidity (RH) below 60% [9]. In addition, there is a concern with packaging that protects museum artifacts and their proper handling [10]. This methodology has attracted most contemporary institutions, once the preventive conservation has developed both theoretical and practical tools, in an interdisciplinary way to support professionals and organizations, avoiding restoration interventions that require more financial resources, and present more risks, especially for textile materials [11,12].

Concerning textile and clothing packaging materials, some requirements must be considered, such as chemical inertness, bearing capacity, precise fit, visual neutrality, strength, and durability. Currently, one of the most used museological packaging materials to separate garments from each other, or involving articles for protection, is raw cotton textile without gum, but it is also noted that this material retains dust due to the textile fabric structure, requiring constant aspiration and additional care. Moreover, this material needs further investigation regarding the relevance of its use, because due to its chemical composition it is susceptible to microbial growth, constituting a carbon source for microorganisms [13,14,15].

Another material that is used is a non-woven polyester fabric, but its composition raises sustainability issues given its difficult subsequent decomposition (plastic waste generator). Moreover, attention should be paid to the choice of a white material, mostly because some institutions used blue tissue paper, and over the years it was discovered that there was a transfer of color to the museum pieces from the paper used [16,17].

It is noticed that museological textiles greatly need protective packaging and these items should be chosen with caution, so it is also essential to investigate whether there are new protection options that can be applied or developed, thinking about this scenario and sustainability issues.

Given these issues, antimicrobial agents of natural origin have attracted the interest of researchers. Essential oils extracted from plants are an interesting possibility, mainly because they can be incorporated into polymeric matrices [18]. Furthermore, the use of the electrospinning technique provides a versatile approach to produce fibers of exceptional length, ranging from 100 nm to several micrometers, with uniform diameter and diverse composition, in which nanofibers are formed from the liquid polymer solution or melt fed through the syringe needle or capillary tube in a high electric field [19,20,21].

In industry there is a system called Nanospider technology from ElMarco company, which allows a scale up of nanofibers, having a technology modified from the traditional electrospinning, and allowing the use of larger volumes of polymer solution deposited in a container. Moreover, instead of using a syringe pump and a needle, cylindrical stainless-steel electrodes are used in constant rotation, and different types of cylindrical electrodes can be chosen according to the desired application [22].

Polymers demonstrate several properties that constitute the electrospun materials, such as strength, weight, porosity, and in some cases even a functionalized surface can be obtained. In particular, synthetic polymers have great advantages over natural ones, related to mechanical properties and rate of degradation, with emphasis on polyurethane (PU) and PCL [23,24,25,26]. Polycaprolactone (PCL) consists of a linear, biodegradable, bioabsorbable, semi-crystalline polyester synthesized by ring-opening polymerization of the ε-caprolactone cycle, and its incorporation with other polymers is widely described in the literature, such as PCL/Polystyrene (PS) incorporated with extract of chamomile oil to inhibit the microorganisms *S. aureus* and *C. albicans* [27,28,29,30].

The incorporation of essential oils extracted from plants has been used in several types of research, due to their aromatic characteristics and medicinal properties, such as antimicrobial, antioxidant, and anti-inflammatory activities [31]. The global essential oils market has grown steadily in recent years, and lavender essential oils have gained prominence contributing to essential oil (EO) production of around 1,500 tons per year. In addition, among the 39 existing species, some studies refer to *Lavandula stoechas luisieri* and *Lavandula luisieri Rozeira* [32].

The EO from *Lavandula luisieri* consists of a liquid with a camphor, fruity, and balsam aroma, and demonstrates antibacterial properties against *Candida albicans* and Gram-positive bacteria, such as *Staphylococcus aureus*, *Sthaphylococcus epidermidis*, and *Streptococcus pyogenes* [33]. Accordingly, our research has focused on the search for more sustainable alternative materials using PCL, and for an antimicrobial material using PCL with EO of *Lavandula luisieri* for application as museological packaging material, aiming to safeguard and preserve items for a longer time for future generations.

The refinement of data allows us to conclude that, despite developments reported so far, the use of *Lavandula luisieri* makes this research work innovative, and to the best of our knowledge this substance has never been explored as an antimicrobial agent for preventing the deterioration of textiles. Moreover, the non-woven substrate based on PCL and *Lavandula luisieri* is revealed to be a promising alternative to the traditional packaging materials used for protection of museological collections.

## 2. Materials and Methods

### 2.1. Materials

Polycaprolactone (PCL) (MW 45.000 g/mol), Mueller Hinton broth (MHB), Tween 80, sodium chloride (NaCl), dimethyl sulfoxide (DMSO), and Resazurin (7-hydroxy-3H-phenoxazin-3-one 10-oxide) dye were bought from Sigma-Aldrich (Sigma-Aldrich, St. Louis, MO, USA). Ethanol absolute and chloroform were purchased from Fisher Chemical (Fisher Scientific, Leicestershire, UK). Nutrient agar (NA), nutrient broth (NB), and agar for microbiology were provided from Fluka (Sigma-Aldrich, St. Louis, MO, USA). *Lavandula luisieri* essential oil (EO) was kindly provided by D’Alenguadiana Company (Dalenguadiana, Mértola, Portugal) located in the South of Portugal.

Textile samples of raw cotton (0.466 mm thickness), and non-woven polyester (0.213 mm thickness) were analyzed as comparative factors for the electrospun materials. Also, a blue cotton fabric (0.568 mm thickness) was used to represent the museum textiles in the contamination test. 

### 2.2. Methods

#### 2.2.1. Chemical Characterization of Essential Oil

The essential oil (EO) of *Lavandula luisieri* was analysed by Gas Chromatography-Mass Spectroscopy (GC-MS) using an Agilent 7890 GC coupled with an Agilent 5975 C inert XL mass selective detector (MSD), operated in the electron impact EI mode (electron energy = 70 eV), scan range = 50–500 amu, and scan rate = 3.99 sans/sec), and an Agilent ChemStation data system. The separation conditions included a DB-5MS fused silica capillary GC column, constituted by a (5% phenyl)-polymethylsiloxane as stationary phase, film thickness of 0.25 mm and an internal diameter of 0.25 mm. The carrier gas was helium, and the column head pressure was 48.7 kPa and a flow rate of 1.0 mL/min. Temperature profile: injector temperature, 250 °C; oven initial temperature, 60 °C for 5 min; temperature rise, 10 °C/min until 250 °C. The GC was operated with an injection volume of 1 mL and a split ratio of 1:50. The identification of components was ascertained by comparison of the retention times and mass spectra with those of the pure standard compounds. All mass spectra were also compared with those of the data system library NIST and Wiley. 

#### 2.2.2. Minimum Inhibitory Concentration (MIC) of the *Lavandula luisieri* Oil

The antimicrobial activity can be evaluated by qualitative and quantitative methods. The qualitative assays commonly consist of agar diffusion tests which are easier to execute and used for subjective evaluations, while the quantitative assays determine the level of bactericidal and fungicidal activity [34,35].

A quantitative method was used to determine the Minimum Inhibitory Concentration (MIC) of *Lavandula luisieri* EO according to the NCLS M07-A6 standard. The test was performed against two bacterial strains *Staphylococcus aureus* ATTC 25933 (*S. aureus*) (Gram-positive) and *Pseudomonas aeruginosa* 27853 (*P. aeruginosa*) (Gram-negative) by the broth microdilution method. Firstly, serial dilutions between 125 µL/mL and 5.81 µL/mL of *Lavandula luisieri* EO were prepared in sterile MHB containing DMSO (10% (*v/v*)). After that, the bacterial suspensions were adjusted to 0.5 McFarland with sterile water, and the working suspensions prepared from 500 µL of 0.5 McFarland and 4500 µL MHB. Then, 50 µL of the working bacterial suspensions and 50 µL of the *Lavandula luisieri* EO dilutions were added to polystyrene 96 multi-well plates (Sigma-Aldrich, St. Louis, MO, USA). The multi-well plates were incubated for 24 h at 37 °C. After incubation, 30 µL of resazurin at a concentration of 0.02% was added to each well of the multi-well plates to aid in the detection of the MIC values. For this purpose, the plates containing resazurin were protected from light and incubated for 4 h. After this period, the wells with the lowest concentration of *Lavandula luisieri* EO which revealed the blue-purple color, indicative of the absence of bacterial growth, were considered the MIC value; color change to pink expressed the presence of bacterial growth. Wells containing the bacterial suspensions in MHB were used as a positive control (K^+^), while wells containing only MHB medium were used as a negative control (K^−^) [36].

#### 2.2.3. Production of the Electrospun Materials

##### Preparation and Electrospinning of the PCL Solution:

In the first phase, a 15% (*w/v*) PCL solution was prepared, dissolving 7.5 g of PCL in 32.5 mL of ethanol and 17.5 mL of chloroform. The PCL solution was kept under magnetic stirring, at a temperature of 40 °C, until complete dissolution of the polymer, during about 30 min.

After that, the pH of the PCL solution was measured in a pH meter with a resolution of 0.1 pH and accuracy of ±0.1 pH, being 7.2. Next, the PCL solution was placed in the electrospinning container with a rotating electrode, and the equipment (Nanospider laboratory machine NS LAB 500S, Elmarco, Liberec, Czech Republic) was programmed according to the following parameters: 80 kV, 55 Hz, 20 °C and with a distance between the rotating electrode and the collector of 13 cm [37].

##### Preparation and Electrospinning of PCL Solution with *Lavandula luisieri* Oil

The PCL solution containing *Lavandula lusieri* EO was prepared following the procedure described above for PCL and 5% owf (over the weight of PCL) of *Lavandula lusieri* EO was added to the solution.

After the PCL + EO solution was prepared, the pH was measured in a pH meter with a resolution of 0.1 pH and accuracy of ±0.1 pH, being 6.6. Finally, the PCL solution containing *Lavandula luisieri* was placed in the electrospinning container, keeping the following parameters fixed: 80 kV, 55 Hz, 20 °C, and 13 cm.

#### 2.2.4. Characterization of the Electrospun Materials’ Morphology

The morphologies of the nanofiber surfaces were analyzed by Scanning Electron Microscopy (SEM). First, samples of PCL (0.304 mm thickness) and PCL with EO (0.309 mm thickness) were assembled onto aluminum SEM stubs with Araldite glue and coated by sputtering (Q150R ES, Quorum Technologies Ltd, Laughton, UK) with a thin layer of gold before analysis. Images were acquired at 5000× magnification using the HITACHI S2700 Scanning Electron Microscope (S2700, Hitachi, Tokyo, Japan), using an accelerating voltage of 20 kV. The mean diameter of the nanofibers and the fiber size distribution were determined by measuring 50 different fibers selected randomly from the SEM micrographs using an image analysis software—Image J (Image J, National Institutes of Health, Bethesda, MD, USA).

#### 2.2.5. Attenuated Total Reflectance-Fourier Transform Infrared Spectroscopy (ATR-FTIR) Analysis

The FTIR was used to confirm the presence of EO in the PCL nanofibers through characteristic absorption bands. The infrared spectra were obtained in the range of 400–4000 cm^–1^ using an ATR-FTIR spectrophotometer (Thermo-Nicolet is10 FT-IR spectrophotometer, Waltham, MA, USA) with an average of 120 scans and a spectral resolution of 4 cm^−1^.

#### 2.2.6. Color Analysis

The spectrophotometric analysis of the textile colors was determined by the Datacolor 110 spectrophotometer (Datacolor 110 spectrophotometer, Datacolor company, Lawrenceville, NJ, USA), which allowed us to characterize the different investigated textiles. The colorimetric values of the textiles were measured using the CIE L*a*b* color system, in which the color coordinates are represented by L* concerning luminosity, while a* expresses the red-green axis (+a indicates red and −a indicates green) and b* represents the yellow-blue axis (+b indicates yellow and −b indicates blue). The results were the average of five color readings for each sample [5]. 

The whiteness index was also determined using the Datacolor equipment and the materials produced by electrospinning (PCL and PCL + EO) were compared with the materials often used in museums (raw cotton and non-woven polyester). The whiteness index was calculated by the following Equation (1):

(1) $$\mathrm{WI}=100-{\left[{\left(100-L^{*}\right)}^{2}+(a^{*}{}^{2}+b^{*2})\right]}^{1/2}$$

#### 2.2.7. Mechanical Analysis

The Young’s modulus, tensile strength, and elongation at break of the materials applied in the museum institutions (raw cotton and non-woven polyester) and those produced in the laboratory (PCL and PCL + OE) were determined using the Adamel Lhomargy DY35 Dynamometer equipment (DY-35, Adamel Lhomargy, Roissy en Brie, France) with a distance between the claws of 10 mm, a 100 N cell at an elongation rate of 5 mm/min, following the EN ISO 13934-1: 2013, in which the results were obtained by an average of 4 measurements for each sample.

#### 2.2.8. Antibacterial Test

The antibacterial activity of PCL and PCL containing EO was evaluated using a quantitative test, following the standard test method E 2180-07 against *S. aureus* and *P. aeruginosa*. For comparative purposes, non-woven polyester and raw cotton were also assessed.

Firstly, a bacterial suspension (1–5 × 10^8^ CFU/mL) was prepared and then added to an agar slurry previously prepared with 0.85% (*w/v*) NaCl and 0.3 (*w/v*) agar-agar in sterile water. After inoculation of the agar slurry, samples of the PCL, PCL + EO, non-woven polyester, and raw cotton, measuring 1.5 × 1.5 cm^2^, were inoculated, evaluated immediately (T_0h_) and after 24 h in contact with the agar slurry at 37 °C (T_24h_). For each sample, serial dilutions were made with 0.85% NaCl (*w/v*), plated on agar plates, and incubated for 24 h at 37 °C. The antimicrobial efficiency was quantitatively expressed as percentage bacterial reduction (%R) using Equation (2), comparing the CFU/mL (Colony Formation Unit) in raw cotton (C) with the CFU/mL in the non-woven polyester (TNT), PCL, and PCL + EO (A) [22].

(2) Percentage Reduction (%R)=((C−A)/C)×100

#### 2.2.9. Contamination Test

**Figure 1:**
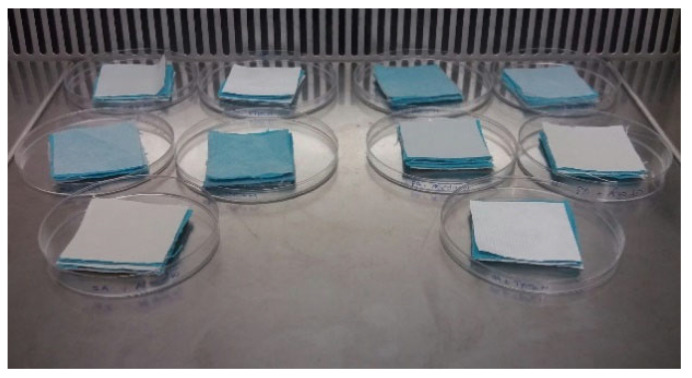
Photo of the contamination test.

Two textile samples (5 × 5 cm^2^) of blue cotton were used, one contaminated with 250 µL of a bacterial suspension of *S. aureus* and *P. aeruginosa* at 0.5 McFarland and another without contamination, as shown in Figure 1. The samples were separated by the chosen material and after 24 h, all textile and material samples were submitted to an antimicrobial test as previously described in the Section 2.2.8 in order to observe if there was passage of bacteria between the layers, as well as the respective CFU/mL. 

## 3. Results and discussion

### 3.1. Chemical Characterization of the Essential Oil

The chemical composition of the EO is presented in Table 1 according to the sequence of elution on a DB5-MS column. A total of 44 components were identified in the EO, corresponding to 91.6% of the total oil. The two main conponents were trans-α-necrodyl acetate (25.8%) and 1,8-cineol (23.3%). The other components present in significant percentages were trans-α-necrodol (10.2%), lavandulyl acetate (5.3%); camphene (3.0%); selina-3,7(11)-diene (2.5%); α-pinene (2.4%), and linalool (2.2%). These eight components correspond to 74.4% of the total oil. 

### 3.2. Minimum Inhibitory Concentration (MIC) of the Lavandula luisieri Oil

**Figure 2:**
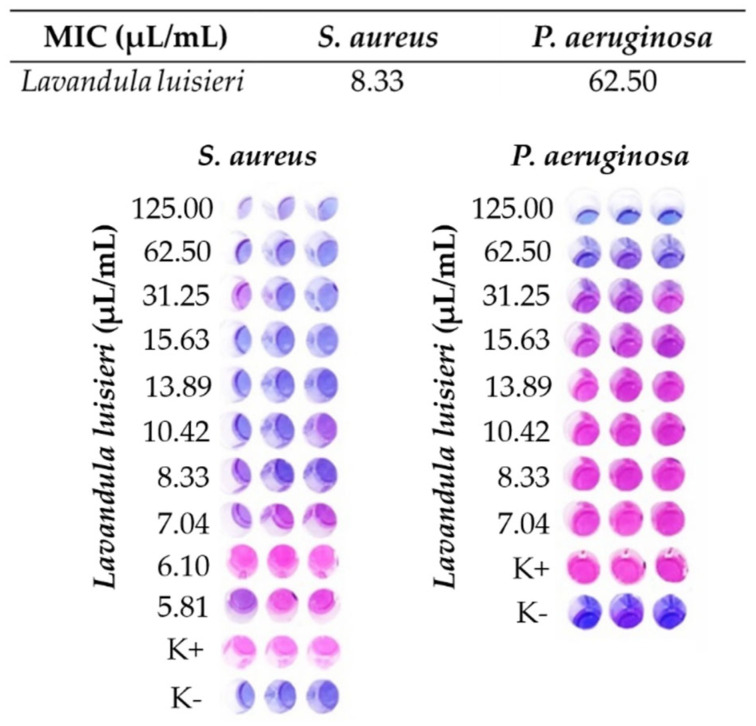
Well plate test and MIC of *Lavandula luisieri* oil.

According to the literature, materials with antimicrobial activity which exhibit biocide properties are able to kill bacteria and fungi, while materials with biostatic properties only inhibit the growth of the microorganisms. The MIC value is the minimum inhibitory concentration necessary for biostatic activity and provides an important information about the amount of the antimicrobial agent required [38]. Figure 2 shows the MIC values obtained for *Lavandula luisieri* against *S. aureus* (8.33 µL/mL) and *P. aeruginosa* (62.50 µL/mL). 

Similar MIC values were described for both *S. aureus* and *P. aeruginosa* in the literature [39]. However, only 5% owf EO, which corresponds to the MIC found for *S. aureus*, was added to the PCL nanofibers, in order to avoid possible interactions between the electrospun packaging materials and the museum items, that could compromise their integrity.

### 3.3. Characterization of the Electrospun Nanofibers’ Morphology

**Figure 3:**
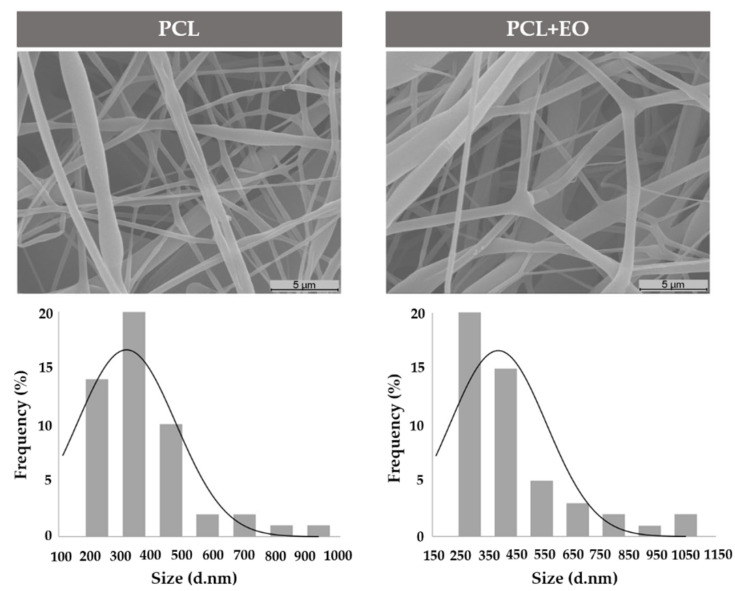
Scanning Electron Microscopy (SEM) images of the nanofibers of the PCL and PCL + EO samples.

The morphology of the PCL and PCL nanofibers with EO can be observed in Figure 3, and the results showed that all samples exhibited a smooth, uniform, and bead-free morphology. The diameter distribution analysis demonstrated a mean diameter for PCL of 318 ± 159.96 nm and for PCL + EO of 366.72 ± 202.58 nm. Moreover, the standard deviation of PCL + EO was higher in comparison with the PCL, which may be due to the incorporation of the *Lavandula luisieri* EO [40].

### 3.4. Attenuated Total Reflectance-Fourier Transform Infrared Spectroscopy (ATR-FTIR) Analysis

**Figure 4:**
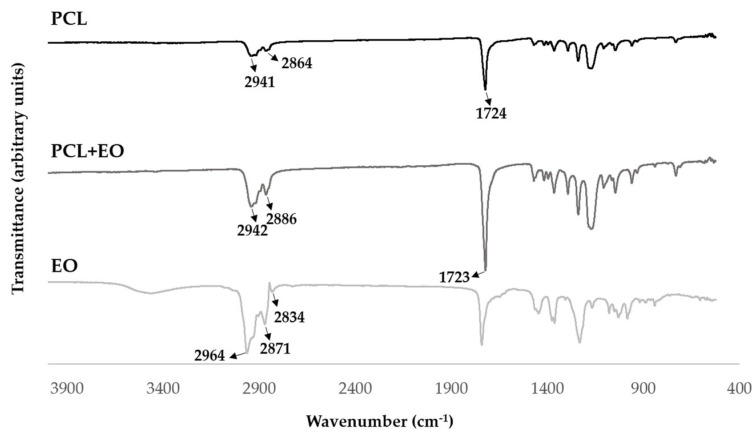
FTIR-ATR analysis of the PCL, PCL + EO, and EO.

The FTIR analysis was performed for the PCL, PCL+EO samples, and EO. The spectra obtained are shown in Figure 4. The PCL spectrum present its characteristic peaks at 2865.22 cm^−1^ and 2943.10 cm^−1^ belonging to the CH_2_ symmetrical and asymmetrical elongation vibration, while the band at 1722.95 cm^−1^ corresponds to the C=O elongation vibration [27].

Concerning the lavender oil extract, the triple division of the C-H elongation peak can be observed in 2964 cm^−1^, 2871 cm^−1^, and 2834 cm^−1^ belonging to the EO. However, the PCL + EO spectrum only showed an increase in the intensity of the bands, once the characteristic peaks of the *Lavandula luisieri* overlapped with the peaks of PCL nanofibers, confirming the effective blending of the EO into the PCL nanofibers [41].

### 3.5. Color Analysis

The color of the materials was analyzed using the Datacolor 110 spectrophotometer. Moreover, the whiteness index was also determined. The whiteness index of a material differs according to the application, and the indexes are used mainly in the textile, paper, and cellulose industry, where the control of whiteness is really important [42].

In this research work, the whiteness index was also an important factor to take into consideration due to the application intended for the electrospun materials produced. Packaging materials for museum applications are required to be white in tone and without any dyes, to easily verify any possible problem with the items stored [15]. Table 2 presents the characterization and spectrophotometric values.

According to the data obtained, the raw cotton exhibited the lowest value of whiteness (16.865 ± 0.684), because this fiber, in its natural form, displays a yellowish hue. On the other hand, bleached cotton is not of interest to museological institutions, since certain reagents used may interact with museological textiles and accelerate their degradation. Therefore, a raw cotton with a neutral pH is required in a museum environment [17].

Concerning non-woven materials, non-woven polyester showed a value of whiteness of 32.253 ± 4.062, and both PCL and PCL with EO presented the best results, corresponding to 93.073 ± 3.398 and 92.751 ± 2.404, respectively. In addition, the PCL with EO displayed a smaller standard deviation.

### 3.6. Mechanical Properties

The tensile strength test was performed on a dynamometer (Adamel Lhomargy DY35). Table 3 presents the results for the raw cotton, non-woven polyester, PCL, and PCL with EO.

The raw cotton textile showed higher tensile strength due to its textile structure. The non-woven polyester also obtained a high value of 4.257 ± 0.337 MPa and high elasticity 25.203 ± 5.822 (%), due to its thermoplastic properties. In turn, the electrospun materials of PCL and PCL with OE displayed values of 1.834 ± 1.640 MPa and 1.139 ± 0.298 MPa, which are in accordance with the data available in the literature [43]. Despite a lower tensile strength, its handling is easy and resistant, being suitable for wrapping textile materials (data resulting from the work—data not shown).

In this context, it is important to remember that the use of PCL is a sustainable alternative, since non-woven polyester is composed of polyester, being a material that requires a long time to be degraded. In addition, the tensile strength of PCL is suitable for museological purposes, similar to silk papers that are also used in the packaging and protection of museological textile pieces.

### 3.7. Antibacterial Efficacy Evaluation

The antibacterial assay on textiles is one of the most important in this research work, because it can demonstrate the antimicrobial effect of the produced packaging materials. The antibacterial assay showed that the growth of bacteria is greater in cotton textiles, which is the most used material in museological packaging. Table 4 shows the results of bacterial reduction in the other materials compared to raw cotton.

The comparison of the materials with the raw cotton showed a bacterial reduction of about 95.22% against *S. aureus* and 94.59% against *P. aeruginosa* for the non-woven polyester. In turn, PCL displayed a lower reduction of 77.51% for *S. aureus* and 86.56% for *P. aeruginosa*. Nevertheless, the PCL + EO showed an efficiency superior to both materials with inhibition rates greater than 99% for both bacteria. These inhibitory values are even higher than other materials with similar EO [41] or other PCL materials with incorporated plant extracts [36]. This result is due to the inherent antimicrobial activity of the *Lavandula luisieri* EO incorporated into the PCL nanofibers. Furthermore, PCL, a synthetic biodegradable polymer, is a more sustainable material and, therefore, could be considered as a future replacement for non-woven polyester. 

### 3.8. Contamination Test

One of the main aims of this study was to confirm that the developed materials behave as protective barriers for museological textiles. In addition, this behavior was analyzed in relation to the materials frequently used in museological packaging. Table 5 shows the results of the contamination test.

It is noticed that all materials behaved as protective barriers preventing bacteria from contaminated textiles from passing to the textiles in the layers above, which is a good result for both PCL and PCL + EO. This is enhanced when durability is also ensured by the developed packaging material [44].

## 4. Conclusions

Museological textiles and clothing are objects of great importance as a cultural and social heritage. The idea of preserving these items is to safeguard something from the past in the present so that in the future it can continue to exist for new generations, and be appreciated or studied in another time [45,46].

This research took place through the dynamics of proving the importance of museological collections, and using the laboratory environment to develop packaging with more sustainable material using PCL and *Lavandula luisieri* EO, in comparison with the existing solutions in the field.

The results achieved showed that raw cotton, despite being one of the most used materials in museological packaging, needs further investigation regarding the relevance of its use, because due to its chemical composition it is susceptible to microbial growth. The so-called TNT proved to be more effective as an antimicrobial barrier, but its polyester composition raises sustainability issues given its difficult subsequent decomposition. Thus, PCL is of interest, and PCL + EO proved to be a potential antimicrobial material for application in museological environments, as it obtained a high bacterial reduction effect.

These findings are new in the research area since it is hard to find specific data for this type of study in the literature. Given our contribution in this study, it is now possible to have a better overview of museological packaging materials, as well as awareness of new sustainable possibilities which we have successfully proved to have an effective antibacterial function and adequate mechanical behavior to assist the museum conservation of textiles.

Thus, our results based on carrying out a test in museums and monitoring the effectiveness of the PCL and PCL + EO developed materials over time with historical textiles and clothing, showed a new pathway to preservation and prevention of contamination of existing museological textiles. The future perspectives of this project consist of carrying out a test in museums and monitoring the effectiveness of the materials developed, namely PCL and PCL + EO, over time with historical textiles and clothing. On the other hand, further studies on the EO loading efficiency will be performed; depending on the textile or clothing to be protected, it will also be possible to optimize the PCL + EO as regards a potential increase in the amount of *Lavandula luisieri* oil used, to adapt and prolong the antimicrobial efficiency over time. The duration of the aromatic properties may also be of interest in this area of conservation. PCL can also be dosed to provide different mechanical properties if they prove to be important for certain articles to be protected. These findings may also be useful for several other applications where bioactive packaging is required.

## Figures and Tables

**Table 1:** Chemical composition of *Lavandula luisieri* oil.

RI	Compound	%
922	1,2,5,5-tetramethyl-1,3-cyclopentadiene	0.1
936	α-Pinene	2.4
950	Camphene	3.0
963	Verbenene	0.1
973	Sabinene	0.1
978	β-Pinene	0.4
989	β-Myrcene	0.1
1015	1,2,3,3-tetramethylcyclopenten-4-one	0.2
1024	*p*-Cymene	0.2
1030	Limonene	0.3
1032	1,8-Cineole	23.3
1038	β-*cis*-Ocimene	0.8
1048	β-*trans*-Ocimene	0.0
1060	γ-Terpinene	0.1
1075	*cis*-Linalool oxide (furanoid)	0.2
1079	3,4,4-trimethylcyclohex-2-en-1-one	0.7
1083	*trans*-linalool oxide (furanoid)	0.2
1085	3,4,5,5-tetramethylcyclopent-2-en-1-one	0.2
1088	Fenchone	0.8
1099	Linalool	2.2
1124	α-Campholenal	0.1
1140	*trans*-Pinocarveol	0.1
1143	Camphor	1.1
1163	*trans*-α-necrodol	10.2
1165	δ-Terpineol	1.2
1168	Lavandulol	1.3
1177	Terpinen-4-ol	0.3
1177	Limonen-4-ol	0.1
1184	*p*-Cymen-8-ol	0.1
1190	α-Terpineol	0.3
1192	Myrtenal	0.1
1206	Verbenone	1.6
1255	Linalool acetate	0.1
1265	*trans*-α-necrodyl acetate	25.8
1289	Lavandulyl acetate	5.3
1376	α-Copaene	0.2
1380	Geranyl acetate	2.0
1387	β-Cubenene	0.1
1453	α-Humulene	0.1
1486	β-Selinene	0.6
1493	α-Selinene	1.0
1523	δ-cadinene	1.1
1541	Selina-3,7(11)-diene	2.5
1637	*t*-Cadinol	1.5

**Table 2:** Characterization and spectrophotometric comparison of produced and control materials.

Spectrophotometric Comparison of Materials				
	Cotton	Non-Woven Polyester	PCL	PCL + EO
Color Appearance	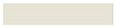	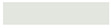	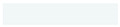	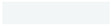
RGB	230.000 227.000 212.000	227.000 230.000 223.000	241.000 246.000 247.000	242.000 246.000 247.000
Reflectance (%/R)	58.046 ± 1.604	52.630 ± 0.711	90.250 ± 2.021	89.052 ± 1.417
Color strength (K/S)	0.152 ± 0.016	0.213 ± 0.009	0.005 ± 0.002	0.007 ± 0.002
L*	91.388 ± 0.386	92.014 ± 0.251	97.618 ± 0.871	97.534 ± 0.528
a*	0.198 ± 0.152	−0.736 ± 0.043	−0.118 ± 0.053	−0.184 ± 0.005
b*	8.846 ± 0.684	3.850 ± 0.092	0.634 ± 0.164	0.926 ± 0.118
Whiteness Index	16.865 ± 0.684	32.253 ± 4.062	93.073 ± 3.398	92.751 ± 2.404

**Table 3:** Characterization and comparison of materials using the tensile strength test. (Data shown as mean ± SD).

	Young’s Modulus (MPa)	Tensile Strength (MPa)	Elongation at Break (%)
Raw Cotton	577.511 ± 57.440	33.759 ± 3.103	5.860 ± 0.446
Non-woven Polyester	17.399 ± 3.006	4.257 ± 0.337	25.203 ± 5.822
PCL	83.547 ± 83.030	1.834 ± 1.640	2.233 ± 0.475
PCL + EO	69.275 ± 22.015	1.139 ± 0.298	1.678 ± 0.228

**Table 4:** Bacterial reduction in materials compared to raw cotton for *S. aureus* and *P. aeruginosa*. Correspondence between percentage of bacterial reduction and the respective CFU/mL. (Values reported as mean ± SD).

	*S. aureus*		*P. aeruginosa*	
	CFU/mL	Bacterial Reduction (%)	CFU/mL	Bacterial Reduction (%)
Raw Cotton	9.03 × 10^7^	-	6.00 × 10^6^	-
Non-woven Polyester	4.32 × 10^6^	95.22%	3.25 × 10^5^	94.59%
PCL	2.03 × 10^7^	77.51%	8.07 × 10^5^	86.56%
PCL+EO	6.02 × 10^5^	99.33%	4.25 × 10^4^	99.29%

**Table 5:** Contamination test result.

	*S. aureus*	*P. aeruginosa*
	CFU/mL	CFU/mL
Control	4.30 × 10^8^	1.01 × 10^8^
Raw Cotton	No growth	No growth
Dyed Cotton	No growth	No growth
Raw Cotton	No growth	No growth
Contaminated Dyed Cotton	1.98 × 10^5^	3.13 × 10^5^
Raw Cotton	No growth	No growth
Non-woven Ppolyester	No growth	No growth
Dyed Cotton	No growth	No growth
Non-woven Polyester	No growth	No growth
Contaminated Dyed Cotton	1.80 × 10^5^	3.92 × 10^6^
Non-woven Polyester	No growth	No growth
PCL	No growth	No growth
Dyed Cotton	No growth	No growth
PCL	No growth	No growth
Contaminated Dyed Cotton	1.52 × 10^6^	2.29 × 10^5^
PCL	No growth	No growth
PCL + EO	No growth	No growth
Dyed Cotton	No growth	No growth
PCL + EO	No growth	No growth
Contaminated Dyed Cotton	8.02 × 10^5^	3.97 × 10^4^
PCL + EO	No growth	No growth

## Data Availability

The data produced in this study are included in the article.
